# A cell-permeable peptide-based PROTAC against the oncoprotein CREPT proficiently inhibits pancreatic cancer

**DOI:** 10.7150/thno.41677

**Published:** 2020-02-19

**Authors:** Danhui Ma, Yutian Zou, Yunxiang Chu, Zhengsheng Liu, Gaochao Liu, Jun Chu, Mengdi Li, Jiayu Wang, Shi-yong Sun, Zhijie Chang

**Affiliations:** 1 State Key Laboratory of Membrane Biology, School of Medicine, National Engineering Laboratory for Anti-tumor Therapeutics, Tsinghua University, Beijing 100084, China.; 2 College of Letters and Science, University of California, Berkeley, 101 Durant Hall, Berkeley, CA 94720.; 3 Department of Gastroenterology, Emergency General Hospital, Beijing 100028, China.; 4 School of Pharmaceutical Sciences, Tsinghua University, Beijing 100084, China.; 5 MOE Key Laboratory of Protein Sciences, Beijing Advanced Innovation Center for Structural Biology, Tsinghua-Peking Joint Center for Life Sciences, School of Life Sciences and School of Medicine, Tsinghua University, Beijing, 100084 China.; 6 Department of Hematology and Medical Oncology, School of Medicine and Winship Cancer Institute, Emory University, Atlanta, GA, USA.

**Keywords:** PROTAC, CREPT, pancreatic cancer, degradation, drug target

## Abstract

Cancers remain a threat to human health due to the lack of effective therapeutic strategies. Great effort has been devoted to the discovery of drug targets to treat cancers, but novel oncoproteins still need to be unveiled for efficient therapy.

**Methods:** We show that CREPT is highly expressed in pancreatic cancer and is associated with poor disease-free survival. CREPT overexpression promotes but CREPT deletion blocks colony formation and proliferation of pancreatic cancer cells. To provide a proof of concept for CREPT as a new target for the inhibition of pancreatic cancer, we designed a cell-permeable peptide-based proteolysis targeting chimera (PROTAC), named PRTC, based on the homodimerized leucine-zipper-like motif in the C-terminus domain of CREPT to induce its degradation *in vivo*.

**Results:** PRTC has high affinity for CREPT, with Kd = 0.34 +/- 0.11 μM and is able to permeate into cells because of the attached membrane-transportable peptide RRRRK. PRTC effectively induces CREPT degradation in a proteasome-dependent manner. Intriguingly, PRTC inhibits colony formation, cell proliferation, and motility in pancreatic cancer cells and ultimately impairs xenograft tumor growth, comparable to the effect of CREPT deletion.

**Conclusions:** PRTC-induced degradation of CREPT leads to inhibition of tumor growth, which is promising for the development of new drugs against pancreatic cancer. In addition, using an interacting motif based on the dimerized structure of proteins may be a new way to design a PROTAC aiming at degrading any protein without known interacting small molecules or peptides.

## Introduction

Cancers are among the deadliest diseases threatening human health 1,2. Conventional therapies for cancers include chemotherapy and radiation therapy, which cause a number of side effects 3-7. To date, molecular targeted therapies have been developed by inhibiting specific pathways, resulting in precise and cytostatic rather than cytotoxic effects 8-11. Conventional targeted therapies include those using small-molecule inhibitors 12-14, monoclonal antibodies 15,16 and RNA interference (RNAi) 17-20.

A novel approach named proteolysis targeting chimeras (PROTACs) emerged in 2001 and showed advantages over conventional targeted therapies 21-30. A PROTAC molecule is a bifunctional chimera consisting of three moieties: a targeting arm, a degradation arm and a linker 31. The targeting arm is a small molecule or peptide that recognizes an intracellular protein as its target. Once a PROTAC molecule interacts with the targeted protein, the degradation arm simultaneously tethers the targeted protein to the E3 ligase and enables its degradation via the proteasome. This technology offers the opportunity to selectively degrade an intracellular protein rather than only to attenuate its activity 32. Currently, PROTAC has been widely utilized to target a variety of proteins, including nuclear receptors, protein kinases and enzymes 33-38. Aberrant expression of these proteins may result in tumorigenesis.

Existing PROTACs are divided into peptide-based PROTACs and small molecule-based PROTACs 38, 39. Peptide-based PROTACs require higher working concentrations than nonpeptidic PROTACs but have several advantages, including facile modification and large protein-protein interaction surfaces, which provide the potential for the development of therapeutic strategies for undruggable oncoproteins with unknown interacting small molecules 38-41.

Cell cycle-related and expression-elevated protein in tumor (*CREPT*, also named *RPRD1B*) was identified by our group as a novel oncogene 42. Elevated CREPT expression was observed in a variety of human tumors 43-46. Mechanistically, CREPT was shown to associate with RNA polymerase II to promote cyclin D1 transcription by inducing chromatin loop formation 42, 47 and activating transcription initiation upon Wnt signaling 48, 49. Other groups have reported that CREPT participates in DNA damage repair 50. We previously showed that CREPT plays a significant role in accelerating cell proliferation and promoting tumorigenesis through the G1 phase 42. Recently, we showed that CREPT is phosphorylated by Aurora B and regulates Cyclin B1 expression to promote G2/M phase transition 51. Accumulating evidence has demonstrated that CREPT is closely related to tumorigenesis 42, 52-55. However, the lack of inhibitors against CREPT restricts the study of the function of this crucial oncoprotein. In this study, we designed a cell-permeable peptide-based PROTAC (named PRTC) to degrade CREPT in pancreatic cancer cells. We showed that PRTC is effective in inhibiting the progression of pancreatic cancer where CREPT is highly expressed and critical for its proliferation.

## Methods

### Plasmids

GST-CREPT, Flag-CREPT and Myc-CREPT were constructed previously in our laboratory 42. Flag-CREPT-RPR and Flag-CREPT-CCT were generated by inserting the PCR-amplified fragments into a pcDNA3.1-Flag vector. To construct pXJ40-GST-CL and pXJ40-GST-CL-m, two pairs of DNA single strand: CL-F (5'-CCGCTCGAGATGAAAGATGTTTTGTCGGAGAAGGAGAAAAAACTAGAGGAATACAAACAGAAGCTTGCACGAGTATGAGGTACCCCG-3'), CL-R (5'-CGGGGTACCTCATACTCGTGCAAGCTTCTGTTTGTATTCCTCTAGTTTTTTCTCCTTCTCCGACAAAACATCTTTCATCTCGAGCGG-3'), CL-m-F (5'-CCGCTCGAGATGAAAGATGTTCCCTCGGAGAAGGAGAAAAAACCCGAGGAATACAAACAGAAGCCCGCACGAGTATGAGGTACCCCG-3'), CL-m-R (5'-CGGGGTACCTCATACTCGTGCGGGCTTCTGTTTGTATTCCTCGGGTTTTTTCTCCTTCTCCGAGGGAACATCTTTCATCTCGAGCGG-3') were synthesized from Beijing Ruibio BiotechCo., Ltd and hereafter generated double strands by gradient freezing. Then we separately inserted DNA fragments into a pXJ40-GST vector.

### Cell Culture and Transfection

AsPc-1, BxPC-3, MIA PaCa-2, HPAC, Panc-1, SW1990, HPDE6-C7 and HEK293T cells were maintained in Dulbecco's modified Eagle's medium (DMEM) supplemented with 10% FBS and 1% penicillin/streptomycin. All cells were cultured at 37 °C with 5% CO_2_. Medium and serum were purchased from Life Technologies (NY, USA). For transient transfection, we used Vigofect (Vigorous Inc. Beijing, China) to transfect plasmids into cells according to the manufacturer's instructions. Panc-1 cells were infected by lentivirus to generate a stable CREPT overexpression cell line. To acquire CREPT deletion cell lines, cells were transfected with the pX458-CREPT-GFP plasmid and screened by flow cytometry.

### Immunohistochemistry (IHC)

The K-ras/p53-driven spontaneous pancreatic cancer mouse model was a kind gift from Professor Feng Qian at Tsinghua University. Mouse pancreatic tumors and normal pancreatic tissues were fixed with 4% paraform and then embedded in paraffin. The pancreatic cancer tissue microarray was purchased from Shanghai Outdo Biotech in China. Immunohistological staining was conducted with an antibody against CREPT according to a protocol performed previously 56.

### CREPT Expression Analysis through Public Data Set

The disease-free survival data on 182 pancreatic cancer samples were downloaded from TCGA database (http://www.cbioportal.org/). The Kaplan-Meier method was used to estimate survival functions for patients with low (n=91) and high (n=91) CREPT expression.

### Co-immunoprecipitation (Co-IP) and Western Blot

For exogenous immunoprecipitation assays, HEK293T cells were plated in 60 mm dishes and then transfected with the indicated plasmids. After 24 h, cells were harvested using RIPA buffer (50 mM Tris-HCl pH 8.0, 2 mM EDTA pH 8.0, 150 mM NaCl, 1% NP-40, 0.5% sodium deoxycholate, 0.1% SDS) with mixed protease inhibitors. Cell lysates were incubated with 2 μg of the indicated antibodies and 30 μL of protein A/G-Sepharose beads at 4 °C overnight. Beads were eluted five times with lysis buffer (50 mM Tris-HCl pH 7.6, 1 mM EDTA pH 8.0, 165 mM NaCl, 0.5% NP-40), and thereafter, a western blot assay was performed to analyze the association by using the indicated antibodies. Western blot was performed as previously reported 56. Anti-β-actin and anti-Flag antibodies were purchased from Sigma. Anti-Myc antibody was purchased from Santa Cruz Biotechnology. Antibody against CREPT was produced in the laboratory 57.

### GST Pull-down

HEK293T cells were plated in 100 mm dishes and then transfected with the indicated plasmids. After 24 h, cells were harvested using RIPA buffer with mixed protease inhibitors. Cell lysates were incubated with 30 μL of GST-tagged Sepharose beads and 10 μg of purified proteins from either *E. coli* or HEK293T cells at 4 °C overnight. Beads were eluted five times with lysis buffer. Western blot was performed using an antibody against GST (Cell Signaling Technology) to analyze the association.

### Reverse Transcriptase PCR (RT-PCR) and Quantitative PCR (qPCR)

Total cellular RNA was extracted using RNeasy Mini Kit (QIAGEN). Real-time PCR was conducted using SuperReal PreMix Plus (TIANGEN Biotech, Beijing, China). Quantitative PCR was conducted using Real-MasterMix (SYBR Green) kit (TIANGEN Biotech, Beijing, China). Primers used for real-time PCR analyses for the human CREPT gene were 5'-CACGCGGGACCCATCGTCTC-3' and 5'-AGCCTTCATCTGCCTCTCTGGCA-3'. Primers used for real-time PCR analyses for the mouse CREPT gene were 5'-AAGATTGCTGAACATCTGGCA-3' and 5'-GTAGTCATCATCTTCCTCCTCTTGT-3'. Gene expression levels were presented as relative values. All the experiments were performed in triplicate.

### Synthesis of Peptides

Peptides used in this research were synthesized by ChinaPeptides Co., Ltd. Crude. All of the peptides were purified by HPLC and confirmed by MS. The evaluation results were shown in the supplementary information.

### Colony Formation

Cells were seeded into 6-well plates (500 cells per well) and cultured for 10 days, then washed with PBS and stained with 0.1% crystal violet. The number of colonies was counted by ImageJ and presented as the mean ± standard deviation (SD) from three individual experiments.

### Cell Viability Assay (CCK-8 Assay)

Cells were seeded into 96-well plates (1000 cells per well) and cultured for the indicated times. Cell viability was evaluated using a CCK-8 kit (Solarbio, Beijing, China). The value of OD450 was calculated by spectrophotometer and presented as the mean ± standard deviation (SD) from three individual experiments.

### Molecular Docking

The CREPT CCT domain structure of CREPT was derived from the Protein Data Bank with accession code 4NAD. A series of rational conformations of PRTC were generated through in silico homology modeling with Schrodinger (Maestro 11.8) and SWISS-MODEL. Then, we chose several 3D linear forms of PRTC and then utilized a constrained protein-protein docking protocol between CREPT CCT domain and PRTC. According to PIPER pose energy and score 58, we picked one of the most reasonable binding poses which was extremely close to classical leucine-zipper-like motif binding pattern.

### Circular Dichroism (CD) Spectroscopy

All peptides were dissolved in the deionized water at a final concentration of 0.1 mg/mL. The CD spectra were measured by using a 1 nm bandwidth with a 1 nm step resolution from 190 to 260 nm at room temperature (Jasco spectropolarimeter, Japan). Final spectra were obtained from the average of three parallel scans after subtracting a spectrum of deionized water recorded under the same conditions. Each sample was scanned thrice and the averaged spectrum was smoothed. The helicity were calculated by CDNN 2.1 59, 60.

### Microscale Thermophoresis (MST)

FITC-labeled PRTC and PRTC-m were regarded as ligands. After a pretest, 100 nM of both PRTC and PRTC-m generated the most appropriate value of fluorescence. Gradient dilutions of purified CREPT proteins were regarded as the target. The Kd value was measured by Microscale Thermophoresis NT.115 and was analyzed by MO.AffinityAnalysis (Nano Temper, German).

### Thermal Shift Assay (TSA)

Purified CREPT proteins were appropriately diluted in a buffer containing 50 mM Tris-HCl, pH 8.0, 150 mM NaCl. The final concentration of CREPT protein was 5.33 μM. SYPRO Orange dye was diluted into 10 μM. The PCR plates were sealed, shaken, and centrifuged after proteins and peptides were added. Thermal scanning (25 to 70 °C at 1 °C/min) was performed using a real-time PCR instrument (Bio-Rad). Melting curves were generated by Prism 7.0 based on the raw data.

### Immunofluorescence Staining

After adherence, cells were treated with 10 μM PRTC for 24 h. The cells were then gently washed three times with warm PBS, fixed with 4% paraform and incubated with 0.25% Triton X-100 in PBST. Next, the cells were blocked in 5% BSA for 1 h at room temperature and incubated with an antibody against CREPT at 4 °C overnight. Cells were washed 3 times with PBST (0.1% Tween-20) and incubated with TRITC-labeled goat anti-mouse IgG antibody (Jackson ImmunoReseach) at a dilution rate of 1:100 at room temperature for 1 h. Coverslips were imaged by a confocal laser scanning microscope (OLYMPUS).

### Wound Healing

Cells were seeded in 6-well plates. Monolayer cells were scratched after culturing to obtain at least 95% confluence. Cells were washed with PBS and maintained in FBS-free DMEM and then treated with 10 μM PRTC and PRTC-m for 24 h. Migration distance was observed by microscopy and was measured by ImageJ from three individual experiments.

### Xenograft Tumor Model

For the xenograft model, 5×10^6^ wild-type Panc-1 cells or CREPT knockout Panc-1 cells were subcutaneously injected into 4-week-old female Balb/c nude mice, which were purchased from Beijing Vital River Laboratory Animal Technology Co., Ltd. Each experimental group consisted of five mice. Mice bearing tumors were randomly divided into two groups and administered via intraperitoneal injection control solvent (0.9% saline) or PRTC (dissolved in 0.9% saline, 10 mg/kg) every 2 days for 4 weeks. The body weight of mice were measured every week after treated with 0.9% saline or PRTC. Tumor volume was measured by using the formula *V* = (*a* × *b^2^*)/2 (*V* is volume, *a* is the length of the tumor, *b* is the width of the tumor).

### Mice and Animal Care

All mice were housed in isolated ventilated cages (maxima six mice per cage) barrier facility at Tsinghua University. The mice were maintained on a 12/12-h light/dark cycle, 22-26 °C with sterile pellet food and water ad libitum. The laboratory animal facility has been accredited by AAALAC (Association for Assessment and Accreditation of Laboratory Animal Care International) and the IACUC (Institutional Animal Care and Use Committee) of Tsinghua University approved all animal protocols used in this study.

### Statistical Analysis

All experiments were repeated at least 3 times. Data were presented as mean +/- standard deviation. Significant differences between groups were determined using a Student's t-test. ****p < 0.0001, ***p < 0.001, **p < 0.01, *p < 0.05.

## Results

### CREPT is highly expressed in pancreatic cancer and promotes cell proliferation

**Figure 1:**
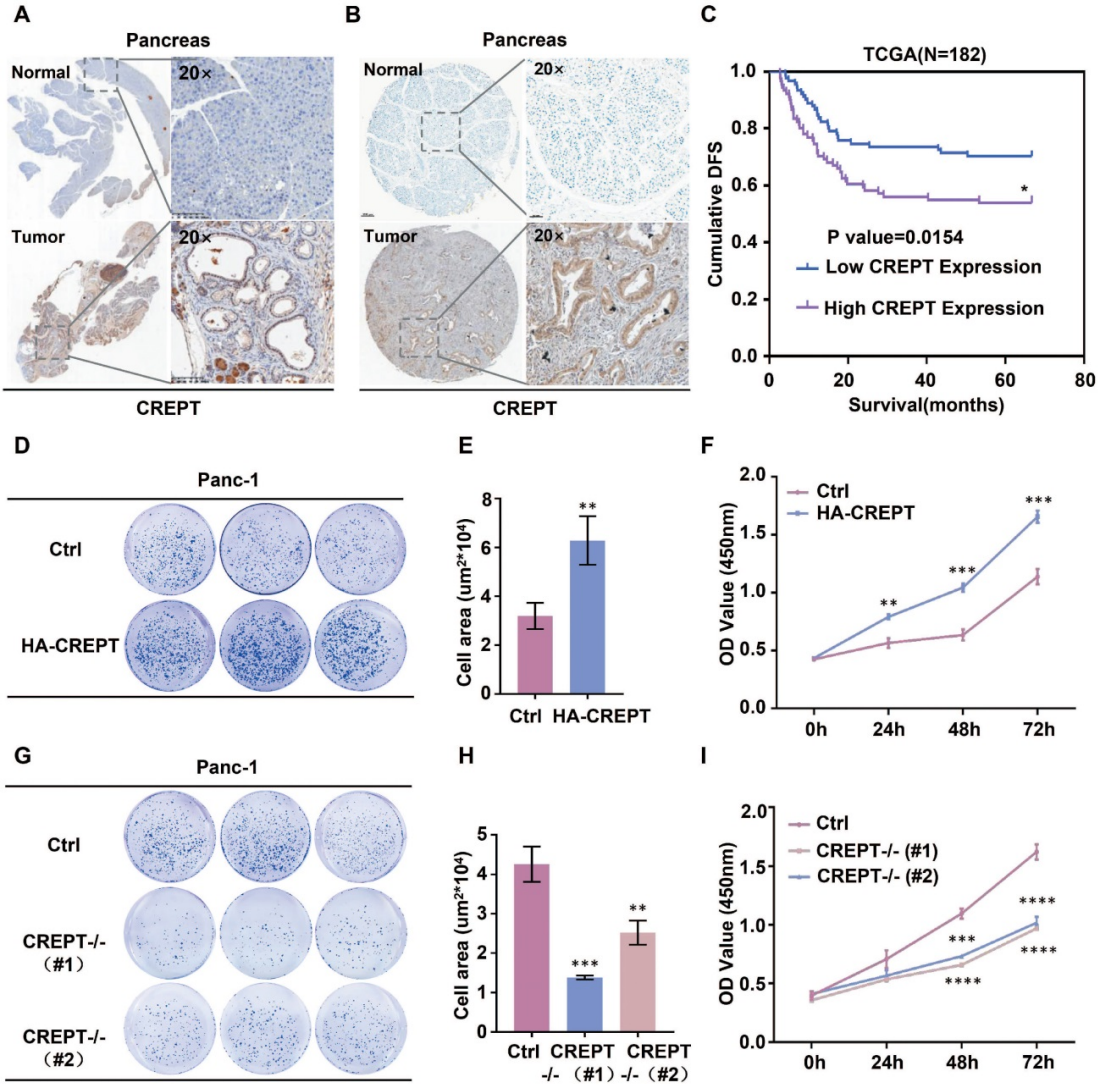
** Overexpression of CREPT in pancreatic cancer promotes cell proliferation and tumorigenesis. (A)** Ectopic expression of CREPT in K-ras/p53-driven pancreatic cancer tissues stained with anti-CREPT antibody. Scale bars, 100 μm. **(B)** Ectopic expression of CREPT in human pancreatic tumor tissues stained with anti-CREPT antibody. Scale bars, 50 μm. **(C)** Kaplan-Meier plot of cumulative disease-free survival (DFS) of 182 pancreatic adenocarcinoma samples in TCGA database. (CREPT high-expression group, purple line; CREPT low-expression group, blue line). The p-value obtained by comparing the two survival curves was 0.0154. **(D-F)** Overexpression of CREPT significantly increased the number of colonies and promoted cell viability. **(G-I)** Deletion of CREPT dramatically reduced the number of colonies and inhibited cell viability. The results are represented as the mean ± SD from three independent repeats.

To address whether CREPT functions in pancreatic cancer, we examined its expression in mouse tissues from K-ras/p53-driven pancreatic cancers and human pancreatic cancer tissues. IHC analyses showed that CREPT is predominantly upregulated in pancreatic cancers in both mouse (Figure 1A) and human (Figure 1B). Western blot and quantitative PCR analyses confirmed that CREPT expression is increased at both the protein and mRNA levels in pancreatic tumors from the mouse (Figure S1A). Increased CREPT expression was also observed in six pancreatic cancer cell lines (Figure S1B). Intriguingly, a high level of CREPT expression was found to be associated with poor disease-free survival in human according to the data from the TCGA database (Figure 1C). These results suggest that CREPT may regulate pancreatic cancer progression. Indeed, stable overexpression of CREPT (Figure S1C) promoted but deletion of CREPT (Figure S1D) inhibited colony formation and cell proliferation in Panc-1 cells, a widely used pancreatic cancer cell line (Figure 1D-I). Taken together, these results suggest that CREPT plays a key role in regulating pancreatic cancer cell proliferation. Therefore, we expect that CREPT might be a candidate target for pancreatic cancer therapy.

### Design and evaluation of PRTC

**Figure 2:**
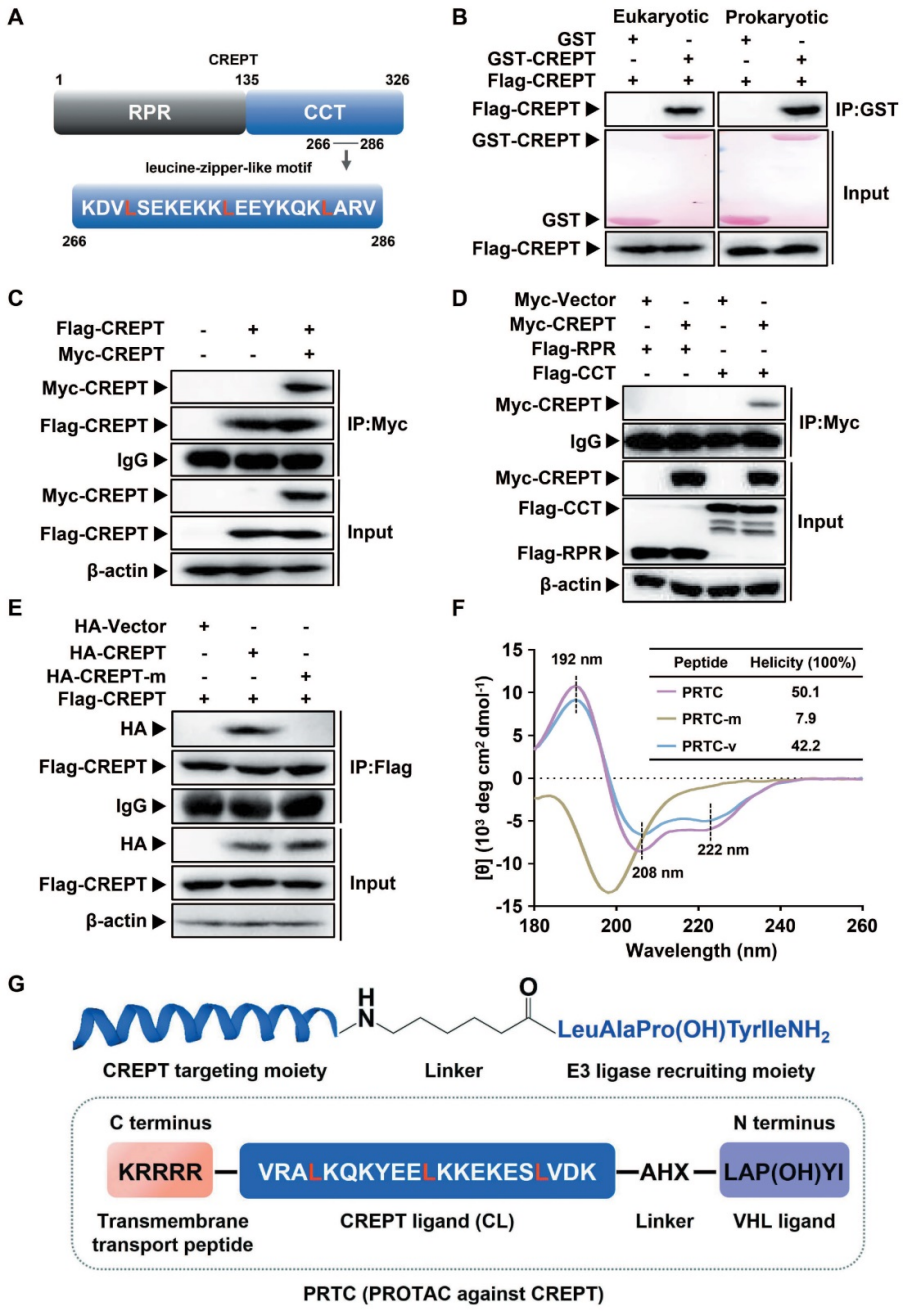
** Design and evaluation of PRTC. (A)** Graphic illustration of the CREPT structure. RPR, regulation of nuclear pre-mRNA. CCT, coiled-coil terminus. **(B)** GST pull-down experiments on Flag-CREPT with purified GST-tagged proteins from HEK293T cells (left) and *E. coli* (right). **(C)** Exogenous IP experiment with Flag-CREPT and Myc-CREPT in HEK293T cells. **(D)** The CCT domain is essential for the formation of homodimers. **(E)** Exogenous IP experiment of Flag-CREPT with HA-CREPT or HA-CREPT-m, which is a mutant in which residues leucine 269, 276, and 283 are replaced with proline residues. **(F)** CD spectroscopy assay of PRTC, PRTC-m and PRTC-v. The positions of 192 nm, 208 nm and 222 nm wavelengths are marked as black dash line. All peptides were dissolved in deionized water at a final concentration of 0.1 mg/mL. **(G)** Schematic diagram of PRTC. CREPT ligand (CL), a polypeptide from amino acids lysine 266 to valine 286. IYP (OH) AL, named VHL ligand. 6-Aminohexanoic acid (AHX) is the linker to bridge the CREPT ligand and VHL ligand. P (OH) is trans-4-hydroxy-L-proline. The pentapeptide (RRRRK) on the C-terminus of PRTC assists PRTC translocation into cells.

To prove the concept of targeting CREPT as a strategy for cancer therapy, we determined to use the PROTAC technology to induce CREPT protein degradation in pancreatic cancer cells. To this end, we designed a PROTAC against CREPT based on its molecular features, hereafter named PRTC. CREPT consists of a N-terminus RPR (regulation of nuclear pre-mRNA) domain and a C-terminus CCT (coiled-coil terminus) domain connecting by a short hinge region. Based on the 3D structure of CREPT CCT domain (PDB code: 4NAD) 61, we found that a leucine-zipper-like motif was located in the C terminus domain from lysine 266 to valine 286 (Figure 2A). This leucine-zipper-like motif contains three XXXLXXX heptads along 21 amino acids, which is a typical α-helix motif for protein homo-dimerization 62-64. Therefore, we predicted that CREPT might form a homodimer 47, 61. Indeed, GST pull-down experiments showed that GST-CREPT purified from mammalian cells or *E. coli* was able to pull down Flag-CREPT (Figure 2B), suggesting that CREPT proteins form homodimers *in vitro*. An immunoprecipitation (IP) experiment further confirmed that CREPT forms a homodimer (Figure 2C). Furthermore, the dimerized CREPT complex was mediated by the CCT domain (Figure 2D). To show the importance of three leucine residues for the dimerization, we generated a mutant (HA-CREPT-m) in which the three leucine residues were replaced with proline residues. An IP experiment showed that HA-CREPT-m failed to interact with Flag-CREPT (Figure 2E), suggesting that three leucine residues are critical for dimerization. Based on this information, we chose the motif (lysine 266 to valine 286, named CREPT ligand, CL) as a targeting arm of PRTC. This motif was connected with the VHL ligand, IYP (OH) AL 65, using 6-aminohexanoic acid (AHX). In addition, to allow better cell permeability, a pentapeptide (RRRRK) was attached to the C-terminus of the PRTC (Figure 2G) 66, 67. The sequence of different variants of PRTC and CL were shown in Table 1.

To further examine whether PRTC remains a helix, we employed circular dichroism (CD) spectroscopy to determine the secondary structure of PRTC, PRTC-m (three leucine residues on PRTC were replaced with proline residues) and PRTC-v (PRTC without the VHL ligand). The CD spectrum of PRTC and PRTC-v showed similar helicity of 50.1% and 42.2% contents, with typical double negative bands at 208 nm and 222 nm and a positive band at 192 nm, demonstrating that PRTC and PRTC-v maintain as α-helix structure. However, PRTC-m exhibited a spectral feature of disordered structure confirming that these three leucine residues on PRTC are essential for the maintenance of the leucine-zipper-like structure (Figure 2F).

### PRTC is able to permeate into pancreatic cancer cells

**Figure 3:**
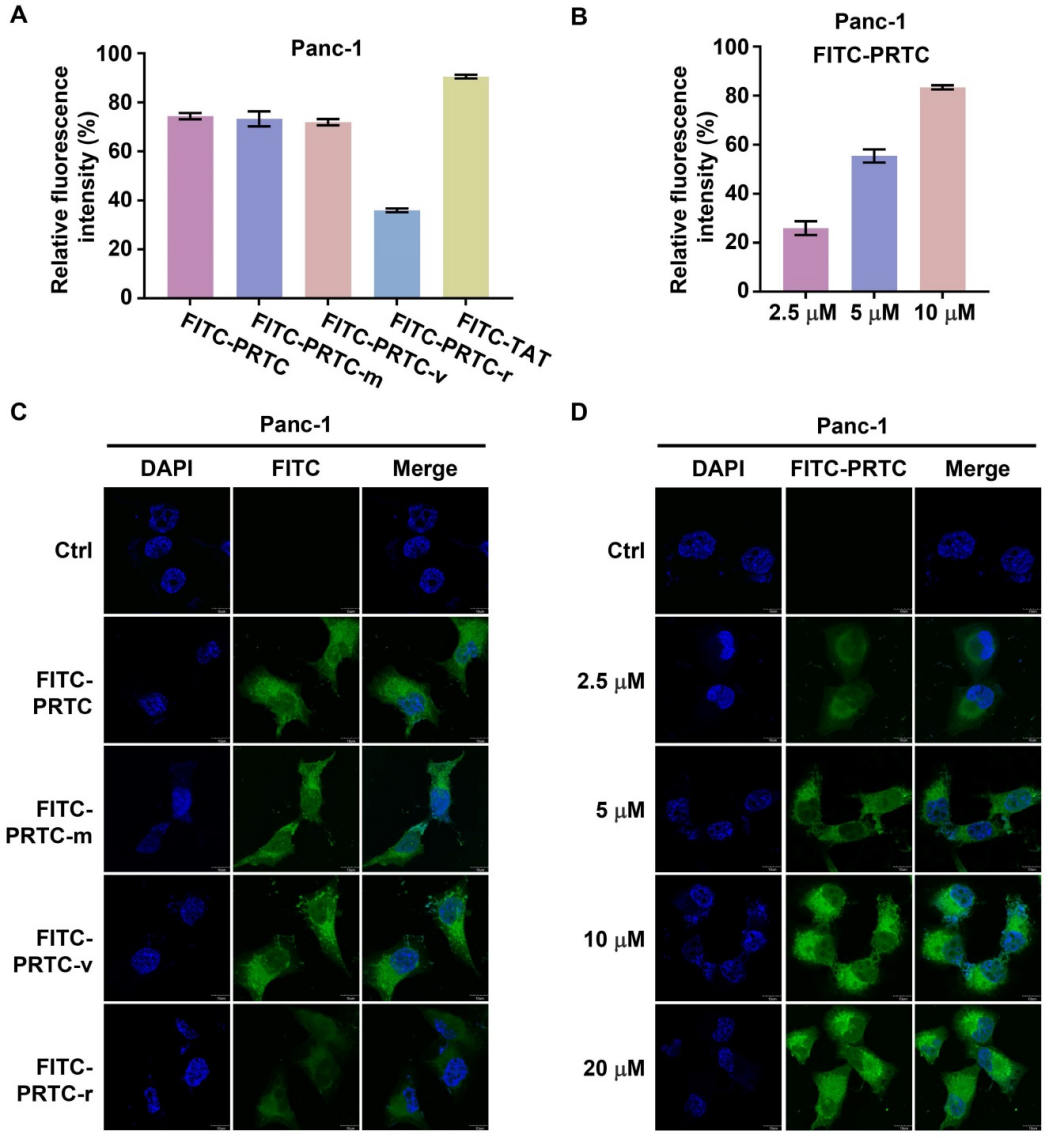
** Identification of the permeability of PRTC in Panc-1 cells. (A)** Entry of FITC- labeled peptides into Panc-1 cells tested by flow cytometry. FITC-TAT is the positive control. Measurements were performed in triplicate. **(B)** Quantification of cellular uptake of Panc-1 cells treated with different concentrations of FITC-PRTC. **(C)** Confocal microscope images of Panc-1 cells treated with 10 μM FITC labeled peptides. Scale bars, 10 μm.** (D)** Confocal microscope images of Panc-1 cells treated with different dosages of FITC-PRTC. Scale bars, 10 μm.

To examine whether PRTC could permeate into pancreatic cancer cells, we synthesized fluorescein isothiocyanate (FITC) labeled PRTC, PRTC-m, PRTC-v, PRTC-r (PRTC without RRRRK) as well as TAT (a widely used cell penetrating peptide, as a positive control). The flow cytometry results showed that the fluorescence intensity in the cells treated with PRTC-r was obviously decreased in comparison with that of cells treated with PRTC, PRTC-m or PRTC-v at 10 μM concentration (Figure 3A). Moreover, the fluorescence intensity in cells treated with different concentrations of FITC-PRTC was significantly increased not only in the Panc-1 cell (Figure 3B) but also in other pancreatic cancer cells including AsPc-1 (Figure S2A) and MIA PaCa-2 (Figure S2B). These results suggest that PRTC has a strong cell permeability due to the transmembrane transport peptide RRRRK. Consistent with the flow cytometry results, fluorescence imaging analyses demonstrated the localization of different FITC labeled peptides in the Panc-1 cells (Figure 3C). Clearly, FITC-PRTC-r showed very weak localization while FITC-PRTC, FITC-PRTC-m and FITC-PRTC-v remained strong localization (Figure 3C, compare bottom panel with other panels). Intriguingly, FITC-PRTC localized into the nucleus, which was clearly observed when the concentration was increased (Figure 3D) and when the incubation time was elongated (Figure S2C). These results were repeated in AsPc-1 and MIA PaCa-2 cells (Figure S2D-S2G). All these results suggest that PRTC permeated into pancreatic cancer cells within 2 h in both dose- and time-dependent manners.

### PRTC associates with CREPT protein

**Figure 4:**
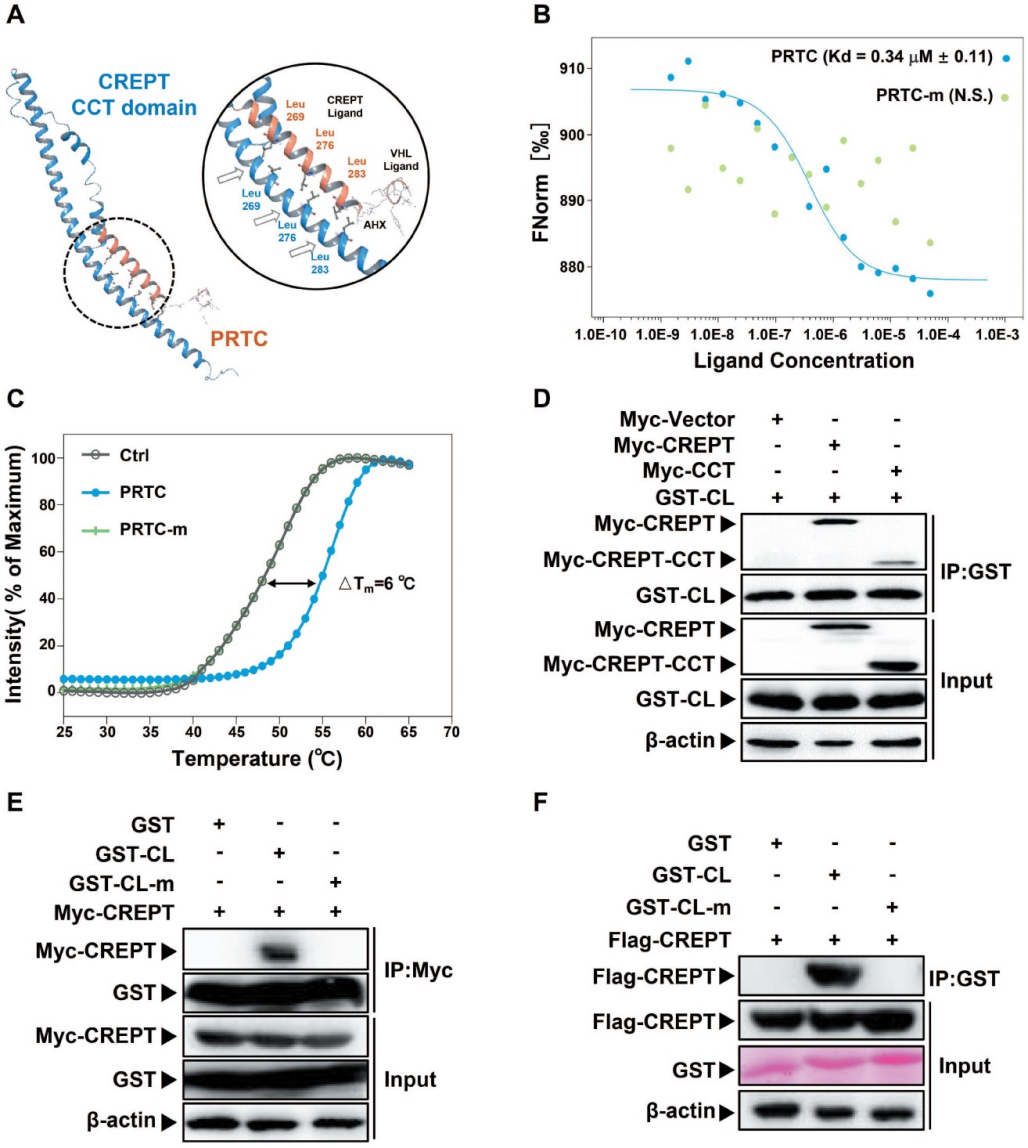
** PRTC is able to interact with CREPT. (A)** Molecular stimulation of the PRTC binding CREPT CCT domain using Schrodinger (Maestro 11.8) and SWISS-MODEL.** (B)** Microscale thermophoresis result of FITC-labeled PRTC with GST-tagged CREPT. Kd = 0.34 ± 0.11 μM. (PRTC, blue dot; PRTC-m, green dot). **(C)** Thermal shift assay results of GST-tagged CREPT with deionized H_2_O (Ctrl), PRTC or PRTC-m. PRTC induced 6 °C shift in T_m_ compared to the control group. **(D)** Exogenous IP experiment of GST-tagged CREPT Ligand (CL) with Myc-CREPT and Myc-CREPT-CCT. **(E)** Exogenous IP experiment of Flag-CREPT with GST-CL or GST-CL-m in HEK293T cells. **(F)** GST pull-down experiments of Flag-CREPT with GST-tagged CL or CL-m using purified proteins from HEK293T cells.

To prove whether PRTC associates with CREPT, we performed a molecular docking to predict possible binding patterns between CREPT CCT domain and PRTC. The simulation result showed that PRTC forms a complex with CREPT CCT domain via three leucine residues (Figure 4A). To examine the predicted simulation result, we performed microscale thermophoresis experiments using FITC-labeled PRTC and purified GST-CREPT protein. The results showed that CREPT associated with PRTC with Kd = 0.34 +/- 0.11 μM, but PRTC-m (leucine 269, 276 and 283 were replaced with proline residues) failed to associate with CREPT (Figure 4B). To further validate PRTC association with CREPT, we performed the thermal shift assay. The results showed that integrated fluorescence value of CREPT protein was shifted by the addition of PRTC (△T_m_ = 6 °C) while the addition of PRTC-m failed to alter the thermal stability of CREPT (Figure 4C). These results suggested that PRTC interacted with CREPT *in vitro*. Then GST protein was tagged with CREPT ligand (CL) for an IP experiment in HEK293T cells. The results showed that GST-CL strongly binds to Myc-CREPT and Myc-CCT (Figure 4D), while GST-CL-m failed to bind to any protein (Figure 4E). Another GST pull-down experiment showed that GST-CL purified from mammalian cells was able to pull down Flag-CREPT *in vitro*, whereas GST-CL-m lost the ability to associate with CREPT (Figure 4F). Taken together, our results demonstrated that the synthesized PRTC is able to associate with CREPT.

### PRTC induces the ubiquitination and proteasome-dependent degradation of the endogenous CREPT protein

**Figure 5:**
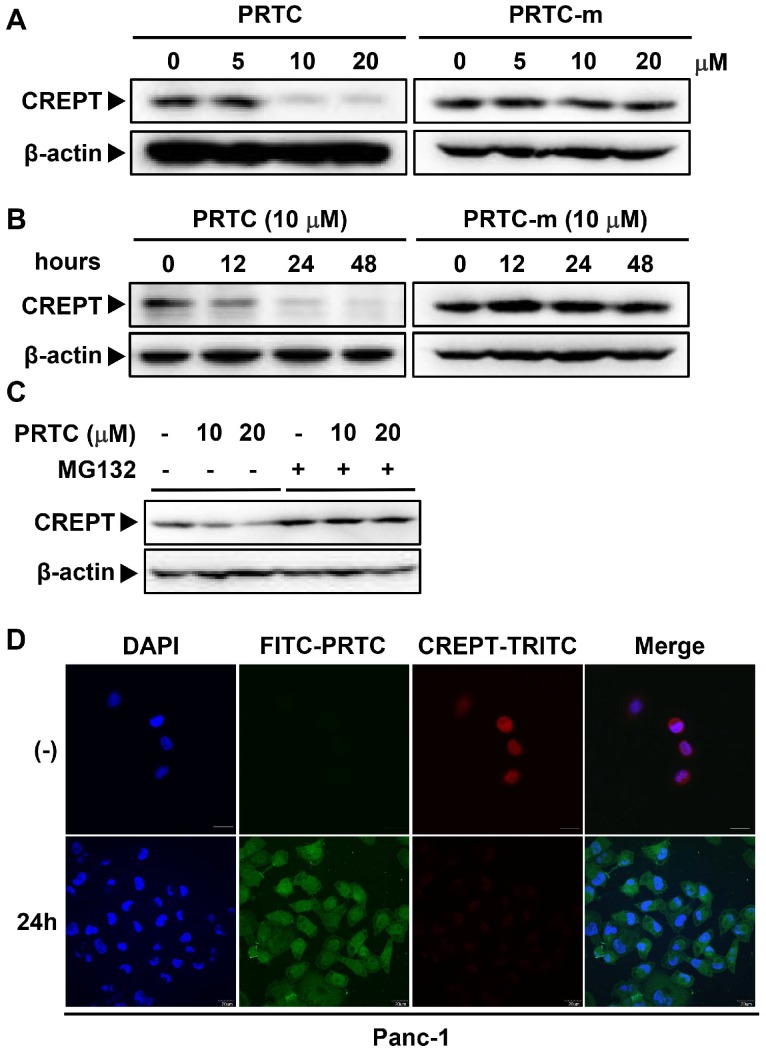
** PRTC induces the ubiquitination and proteasome- dependent degradation of the endogenous CREPT protein. (A)** Immunoblot of CREPT following 24 h incubation with different dosages of PRTC or PRTC-m in Panc-1 cells. **(B)** Immunoblot of CREPT exposed to 10 μM PRTC or PRTC-m for different times in Panc-1 cells. **(C)** Western blot analysis of CREPT following 24 h incubation with 10 μM PTRC in Panc-1 cells. MG132 is an inhibitor of proteasome activity. **(D)** Immunofluorescence visualization of Panc-1 cells after treatment with PRTC for 24 h. Cells were stained with anti-CREPT antibody. Scale bars, 20 μm.

To address whether PRTC could induce CREPT degradation, we examined the endogenous protein level of CREPT in Panc-1, AsPc-1 and MIA-PaCa-2 cells in the presence of different dosages of PRTC. Western blot analyses showed that CREPT protein was dramatically decreased upon the addition of PRTC, but not PRTC-m, with the DC_50_ value of 10 μM (Figure 5A, Figure S3B-3C). Consistently, treatment with PRTC led to decreases in CREPT protein at different times (Figure 5B, S3A). Of note, PRTC-m showed no effect on the CREPT protein levels at different time points (Figure 5B, S3A, right panels). To prove that the decreased levels of CREPT protein were due to ubiquitin-induced proteasome degradation, we treated the cells with MG132, an inhibitor of proteasome activity. The results showed that the addition of MG132 completely abolished the PRTC-induced CREPT degradation (Figure 5C). To further visualize the degradation process, we stained the endogenous CREPT protein with an antibody against CREPT in the cells treated with FITC-labeled PRTC. The results showed that FITC-PRTC permeated into the cells at 1.5 h and started to induce the degradation of TRITC-CREPT in Panc-1 cells at 12h (Figure S3D), and almost completely depleted the endogenous CREPT protein at 24 h (Figure 5D). All these results suggest that PRTC is able to induce the degradation of endogenous CREPT protein in dose- and time-dependent manners.

### The degradation of CREPT by PRTC is dependent on its VHL ligand

**Figure 6:**
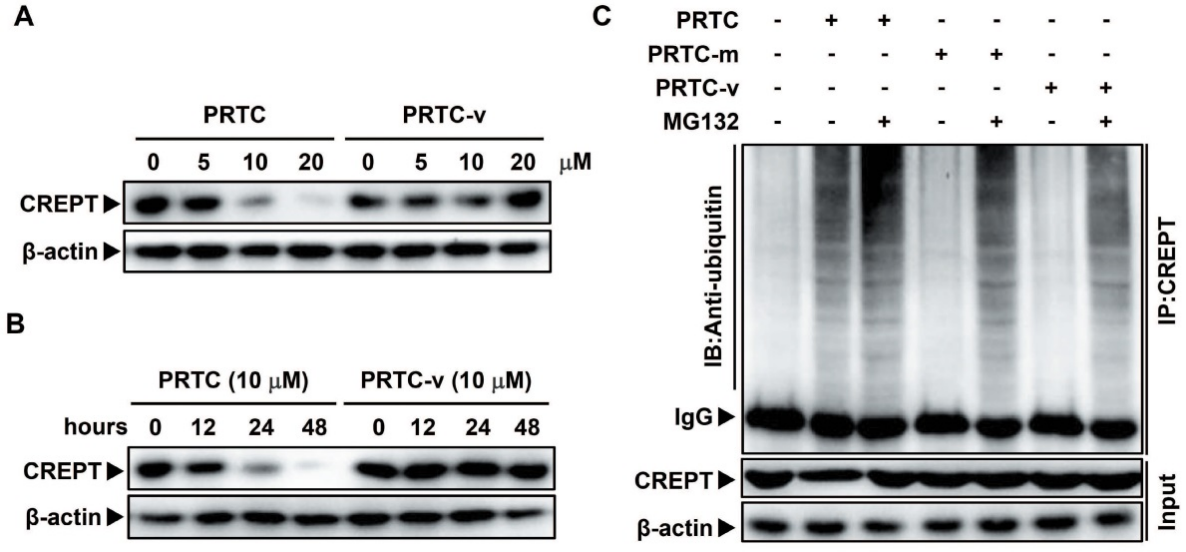
** The degradation of CREPT by PRTC is dependent on its VHL ligand. (A)** Immunoblot of CREPT following 24 h incubation with different dosages of PRTC or PRTC-v in Panc-1 cells. **(B)** Immunoblot of CREPT exposed to 10 μM PRTC or PRTC-v for different time points in Panc-1 cells. **(C)** Endogenous IP experiment of CREPT with ubiquitin treated with 10 μM PTRC, PTRC-m or PTRC-v in Panc-1 cells. IP: CREPT, IB: Anti-ubiquitin.

To further demonstrate that the degradation of CREPT is mediated by PRTC, Panc-1 cells were treated with PRTC or PRTC-v for different dosages or time points. Western blot results showed that PRTC-v failed to degrade endogenous CREPT protein due to the lack of the VHL ligand (Figure 6A-B). In addition, a Co-IP experiment was performed in Panc-1 cells treated with 10 μM PRTC, PRTC-m or PRTC-v for 24 h. The results showed that CREPT protein level was significantly decreased, accompanied with increased ubiquitinated CREPT proteins in the presence of PRTC. In the absence of MG132, the level of ubiquitinated CREPT was further increased by PRTC (Figure 6C, compare lane 1 and lane 2). However, both PRTC-m and PRTC-v were unable to mediate the accumulation of ubiquitinated CREPT (Figure 6C, compare lane 1, lane 4 and lane 6). Taken together, we demonstrated that the VHL ligand of PRTC is responsible for proteasome-dependent degradation of CREPT.

### PRTC inhibits cell proliferation and tumorigenesis in pancreatic cancer

**Figure 7:**
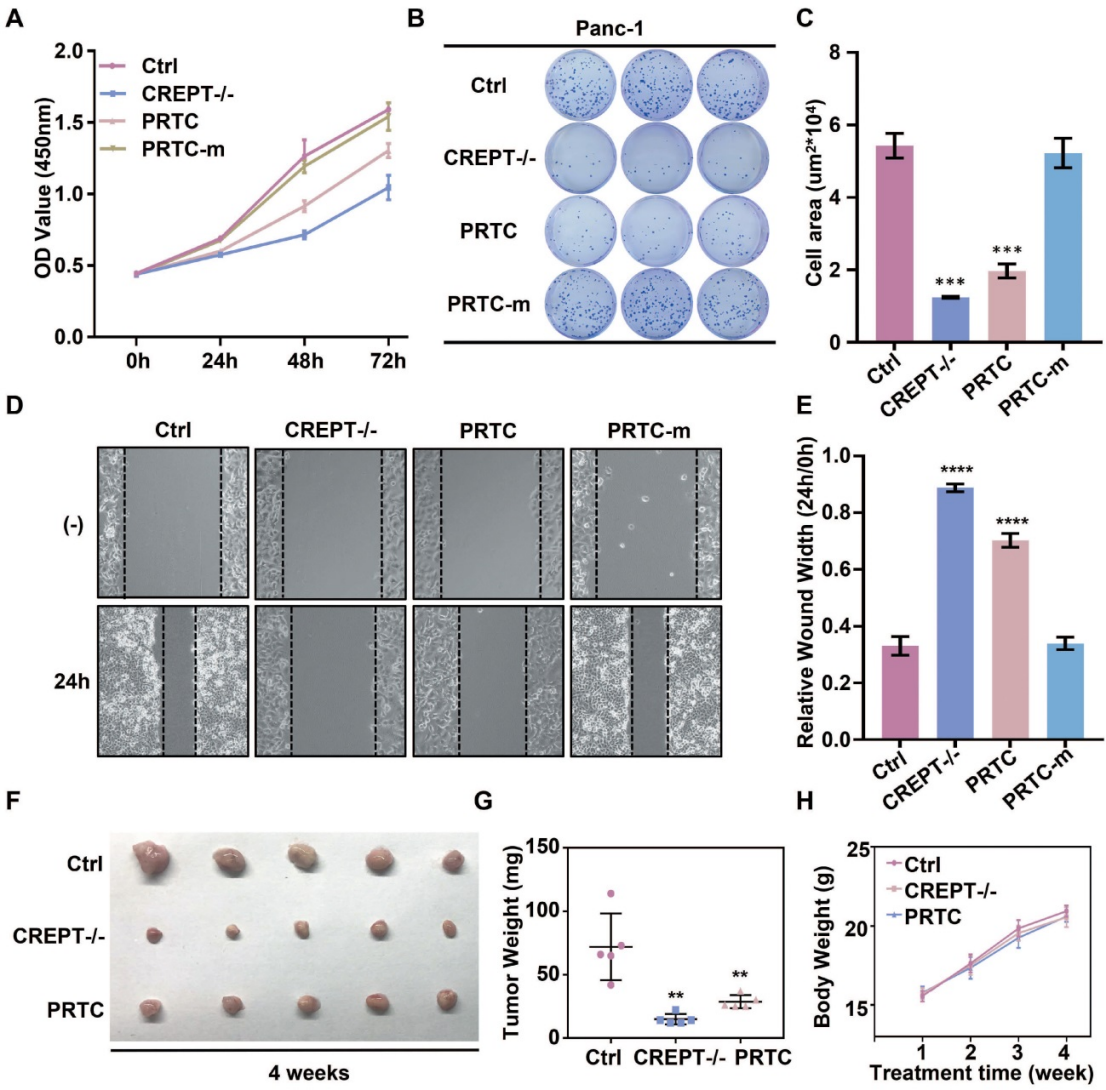
** CREPT degradation by PRTC treatment inhibits cell proliferation and tumorigenesis in pancreatic cancer. (A)** CCK-8 assay of wild-type Panc-1 cells treated with deionized H_2_O (Ctrl), CREPT deletion cells treated with deionized H_2_O (CREPT-/-), Panc-1 cells treated with 10 μM PRTC (PRTC) and Panc-1 cells treated with 10 μM PRTC-m (PRTC-m). **(B-C)** Colony formation of Ctrl, CREPT-/-, PRTC and PRTC-m. **(D-E)** Wound healing assay of Ctrl, CREPT-/-, PRTC and PRTC-m. **(F)** Xenograft tumor formation of Ctrl, CREPT-/-, PRTC group. 5×10^6^ wild-type Panc-1 cells or CREPT knockout Panc-1 cells were subcutaneously injected into 4-week-old female Balb/c nude mice. Mice bearing tumors were randomly divided into two groups and intraperitoneally administered control solvent (0.9% saline) or PRTC (10 mg/kg) every 2 days for 4 weeks. **(G)** Tumor weights of xenograft tumors. **(H)** Body weight curves of Ctrl, CREPT-/-, PRTC group. n=5. The values were measured every week after treated with 0.9% saline or PRTC.

To address whether the PRTC-induced degradation of CREPT is critical for the inhibition of cell proliferation and tumor growth, we performed cell proliferation and colony formation experiments in Panc-1 cells in the presence of PRTC and PRTC-m. We used a CRISPR-induced CREPT deletion cell line as a control to demonstrate the efficiency of PRTC. The results showed that deletion of CREPT and addition of 10 μM PRTC dramatically inhibited cell proliferation, but PRTC-m (Figure 7A) and PRTC-v (Figure S4C and S4F) failed. The PRTC-treated cells yielded fewer colonies than the control cells, similar to the effect of CREPT deletion (Figure 7B-C, Figure S4A-B and S4D-E). A wound healing assay demonstrated that PRTC, but not PRTC-m, decreased cell movement, comparable to the effect of CREPT deletion (Figure 7D-E). These results suggest that the degradation of CREPT by PRTC could inhibit tumor cell proliferation, colony formation and migration. To further address whether PRTC has an antitumor activity *in vivo*, we administered PRTC intraperitoneally in mice with xenograft tumors. The results showed that the tumor weight and size were significantly decreased after treatment with PRTC for 4 weeks in comparison with those in the control mice, although the difference was slightly weaker than that in the CREPT deletion group (Figure 7F-G and Figure S4G). The body weight of the mice administrated with PRTC was at the similar levels to the control mice (Figure 7H). Furthermore, we found that PRTC failed to inhibit the cell proliferation of HPDE6-C7 cells, a normal pancreatic epithelial cell line with little CREPT expression (Figure S4H). These results demonstrated that the toxicity of PRTC is relatively low. Taken together, these results show that PRTC induces CREPT degradation and thereafter inhibits tumor growth *in vivo*.

## Discussion

**Figure 8:**
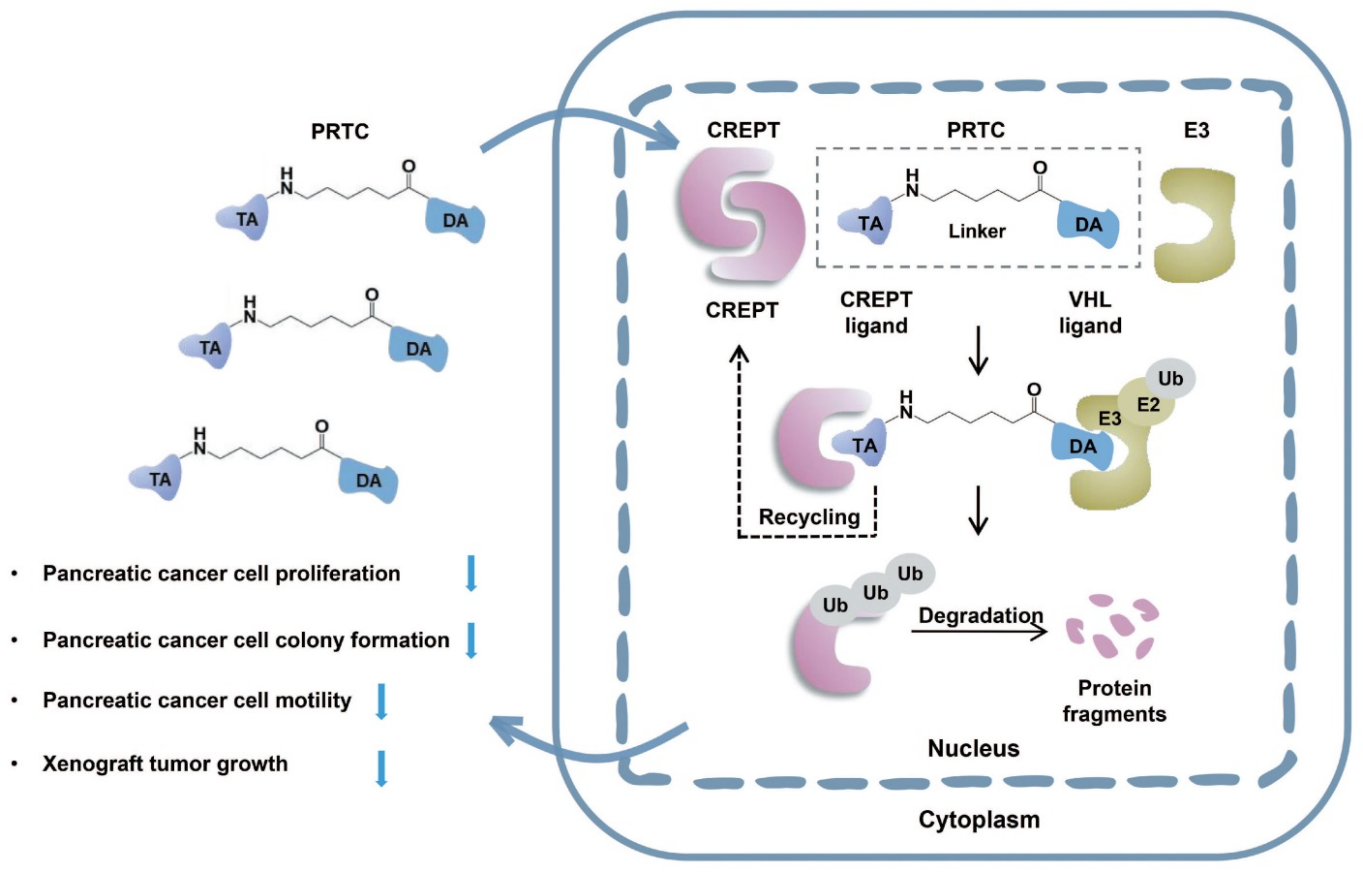
** Schematic diagram of PRTC degrading the oncoprotein CREPT.** PRTC permeates into pancreatic cancer cells and competitively binds dimerized CREPT. Meanwhile, PRTC binds an E3-ubiquitin ligase complex. The formation of the trimeric complex contributes to the transfer of ubiquitins to the CREPT. The poly-ubiquitinated CREPT is recognized by the proteasome and degraded. PRTC proficiently inhibits colony formation, cell proliferation, and motility in pancreatic cancer cells and ultimately impairs xenograft tumor growth. TA, the targeting arm of PRTC. DA, the degrading arm of PRTC. Ub, ubiquitin.

In this study, we take advantage of the proteolysis targeting chimera (PROTAC) technology to induce CREPT protein degradation in pancreatic cancer cells. CREPT consists of an RPR domain and a CCT domain connecting by a short hinge region 47, 61. The crystal structure of both RPR and CCT domains were resolved, however, full-length CREPT remains no structure recognition due to the highly flexible and tender hinge region. Based on the 3D structure of CREPT CCT domain, we chose a dimerized leucine-zipper-like motif (lysine 266 to valine 286) located in the CCT domain as the targeting arm for the PROTAC design. This is a new motif and no attempt was made to use this motif as a target for CREPT. In this study, we used this motif to tether the VHL ligand for our PROTAC molecule construction. To increase cell permeability, a pentapeptide (RRRRK) was attached to the C-terminus of the PROTAC molecule. Both flow cytometry and fluorescence results demonstrated our designed PROTAC against CREPT (named PRTC) is able to translocate into the cell through permeating the cell membrane and induces degradation of endogenous CREPT protein in pancreatic cancer cells. We have provided evidence that PRTC worked as a suitable molecule to target CREPT as a proof-of-concept for the development of new drugs against cancers (Figure 8).

The CD spectrum illustrates that PRTC mainly consists of one α-helix, which is consistent with the prediction of molecular docking study. This α-helix of PRTC could be able to form a complex with the extended α-helix in the CREPT protein. Theoretically, two extended α-helixes in the CREPT proteins could form a more stable complex than that from one short α-helix with one extended α-helix. We prospect that a larger amount of PRTC molecules competitively bind to CREPT monomer and subsequently tether to the E3 ligase for proteasome-dependent degradation (Figure 8). In this case, the dimerized CREPT complex could be interrupted by the short α-helix of PRTC. While PRTC per se remains un-degraded 31, the amount of endogenous CREPT dimers starts to decrease by degradation. Therefore, PRTC is very effective on the degradation of endogenous CREPT proteins, no matter how CREPT remains of monomer or dimer form.

PRTC effectively induced the degradation of CREPT, resulting in the inhibition of tumor cell proliferation, colony formation and migration. To demonstrate that the observed cell proliferation inhibition of three pancreatic cancer cells is due to CREPT degradation rather than the disruption of the CREPT homodimers caused by the non-degrading peptide, we synthesized a PRTC mutant lacking the VHL recruiting domain, named PRTC-v. Intriguingly, PRTC-v restrained the ability to degrade endogenous CREPT protein in proteasome-dependent manner due to the lack of the VHL ligand. Furthermore, we observed that the addition of PRTC-v failed to inhibit cell proliferation. These functional studies suggested that targeting CREPT for degradation by PRTC is the main mechanism of this molecule on inhibition of CREPT protein activity. This also confirmed our expectation that simply disassociation of endogenous CREPT dimers has less effect on its activity.

The design of PROTACs remains a challenge due to the lack of an effective targeting arm for targeted proteins 68. Previous studies mainly focused on using a small molecule or a peptide that interacts with the targeted protein 26, 29. However, it is difficult to screen or design a small molecule or peptide that interacts strongly with a protein. In this study, we proposed that using a motif responsible for dimerization could be effective for designing the targeting arm of PROTACs. We expect that selecting a motif responsible for the protein complex formation could be used to design targeting arm for other proteins without known interacting small molecules or peptides for PROTACs. Our study extended the applications of the PROTAC technology through degrading “undruggable” proteins. With this proof-of-concept model, we provided evidence that PRTC significantly decreases the tumor size and weight in mice. Intriguingly, we observed no toxicity of PRTC in the treatment of mice bearing xenograft tumors. This study offers an opportunity for drug development for pancreatic cancer therapy based on the highly expressed oncoprotein CREPT.

## Figures and Tables

**Table 1:** Sequence of peptides used in this research.

Peptides	Sequence (N-C)
PRTC	IYP (OH) AL-AHX-KDVLSEKEKKLEEYKQKLARV-RRRRK
PRTC-m	IYP (OH) AL-AHX-KDVPSEKEKKPEEYKQKPARV-RRRRK
PRTC-v	KDVLSEKEKKLEEYKQKLARV-RRRRK
PRTC-r	IYP (OH) AL-AHX-KDVLSEKEKKLEEYKQKLARV
FITC-PRTC	FITC-AHX-IYP (OH) AL-AHX-KDVLSEKEKKLEEYKQKLARV-RRRRK
FITC-PRTC-m	FITC-AHX-IYP (OH) AL-AHX-KDVPSEKEKKPEEYKQKPARV-RRRRK
FITC-PRTC-v	FITC-AHX-KDVLSEKEKKLEEYKQKLARV-RRRRK
FITC-PRTC-r	FITC-AHX-IYP (OH) AL-AHX-KDVLSEKEKKLEEYKQKLARV
CL (CREPT Ligand )	KDVLSEKEKKLEEYKQKLARV
CL-m (CREPT Ligand-m)	KDVPSEKEKKPEEYKQKPARV
VHL Ligand	IYP (OH) AL
Transmembrane transport peptide	RRRRK

For the peptides, the blue part was the sequence targeting CREPT. The black part was the linker group. The purple part was the sequence binding VHL E3 ubiquitin ligase. The orange part was the sequence permeating cell membrane. For flow cytometry and fluorescence assays, fluorescein isothiocyanate (FITC) was labeled at the N-terminus with a AHX linker. AHX, 6-aminohexanoic acid. P(OH), trans-4-hydroxy-L-proline.
