# Supplementary material for: A cell-permeable peptide-based PROTAC against the oncoprotein CREPT proficiently inhibits pancreatic cancer
Source: Theranostics. 2020 Feb 19;10(8):3708–21. doi: 10.7150/thno.41677 (PMC7069095; doi:10.7150/thno.41677)
Supplement: Supplementary file 1 — Supplementary figures. [file thnov10p3708s1.pdf]

SUPPLEMENTARY INFORMATION

A cell-permeable peptide-based PROTAC against the oncoprotein CREPT proficiently inhibits pancreatic cancer

Danhui Ma[1], Yutian Zou[1, 2], Yunxiang Chu[3], Zhengsheng Liu[4], Gaochao Liu[5], Jun Chu[1], Mengdi Li[1], Jiayu Wang[1], Shi-yong Sun[6, *], Zhijie Chang[1, *]

1. State Key Laboratory of Membrane Biology, School of Medicine, National Engineering Laboratory for Anti-tumor Therapeutics, Tsinghua University, Beijing 100084, China.
2. College of Letters and Science, University of California, Berkeley, 101 Durant Hall, Berkeley, CA 94720.
3. Department of Gastroenterology, Emergency General Hospital, Beijing 100028, China.
4. School of Pharmaceutical Sciences, Tsinghua University, Beijing 100084, China.
5. MOE Key Laboratory of Protein Sciences, Beijing Advanced Innovation Center for Structural Biology, Tsinghua-Peking Joint Center for Life Sciences, School of Life Sciences and School of Medicine, Tsinghua University, Beijing, 100084 China.
6. Department of Hematology and Medical Oncology, Emory University School of Medicine and Winship Cancer Institute, Atlanta, GA, USA.

*Correspondence: Zhijie Chang, Ph.D. E-mail: zhijiec@mail.tsinghua.edu.cn. or Shi-yong Sun, Ph.D. E-mail: ssun@emory.edu.

1. Abbreviations

AHX, 6-aminohexanoic acid
CCK-8, cell counting kit
CCT, coiled-coil terminus
CD, circular dichroism
CL, CREPT ligand
CREPT, cell-cycle-related and expression-elevated protein in tumor
DA, degrading arm
DAPI, 4′, 6-diamidino-2-phenylindole
DC_{50}, half maximal degradation concentration
DMEM, Dulbecco's modified Eagle's medium
EDTA, ethylenediaminetetraacetic acid
FBS, fetal bovine serum
FITC, fluorescein isothiocyanate
GST, glutathione S-transferase
HPLC, high performance liquid chromatography
IHC, immunohistochemistry
IP, immunoprecipitation
Kd, dissociation constant
MS, mass spectrum
MST, microscale thermophoresis
OD, optical density
PAAD, pancreatic adenocarcinoma
PBS, phosphate buffer saline
PFA, paraformaldehyde

PROTAC, proteolysis targeting chimeras
PRTC, a PROTAC against CREPT
qPCR, quantitative PCR
RNAPII, RNA polymerase II
RNAi, RNA interference
RPR, regulation of nuclear pre-mRNA
RT-PCR, reverse transcriptase PCR
TA, targeting arm
TCGA database, the cancer genome atlas database
TRITC, tetramethylrhodamine
TSA, thermal shift assay
VHL, von hippel-lindau

2. Evaluation of synthesized peptides

1) FITC-PRTC: 5-FITC-(Ahx)-IY-(Hyp)-AL-(Ahx)-KDVLSEKEKKLEEYKQKLARVKRRRR

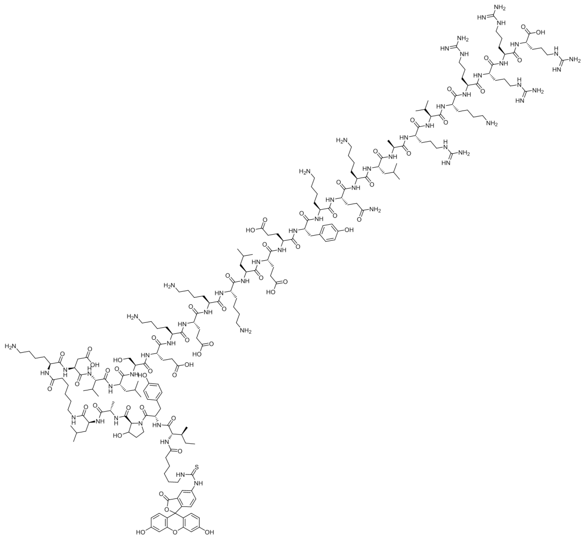

HPLC report:

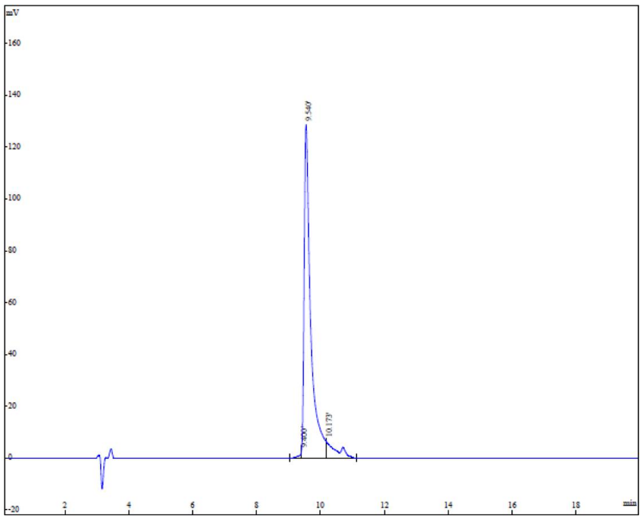

Peak No.	Ret Time	Area	Conc.
1	9.400	15367	0.7737
2	9.540	1819411	91.6

3	10.173	151321	7.619
**Total:**		**1986099**	**100.0000**

MS report:

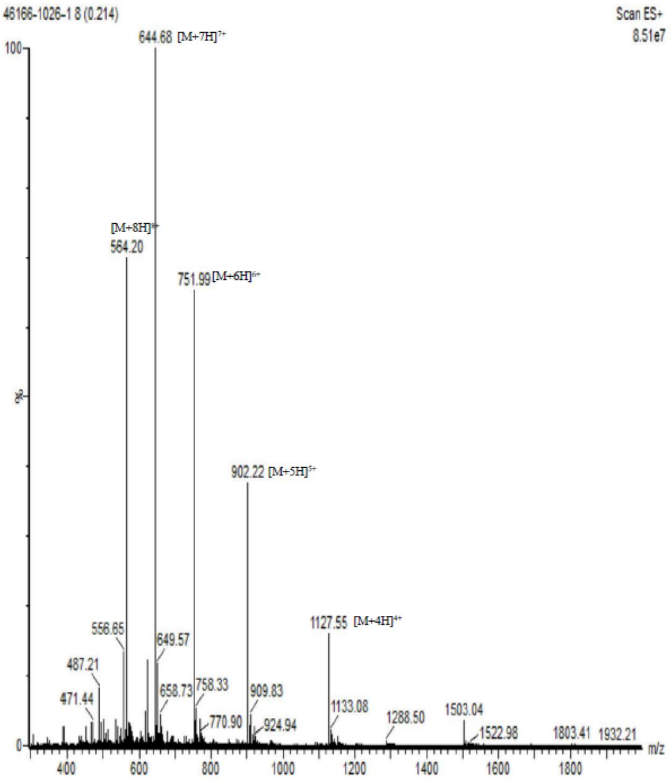

2) FITC-PRTC-m: 5-FITC-(Ahx)-IY-(Hyp)-AL-(Ahx)-KDVPSEKEKKPEEYKQKPARVKRRRR

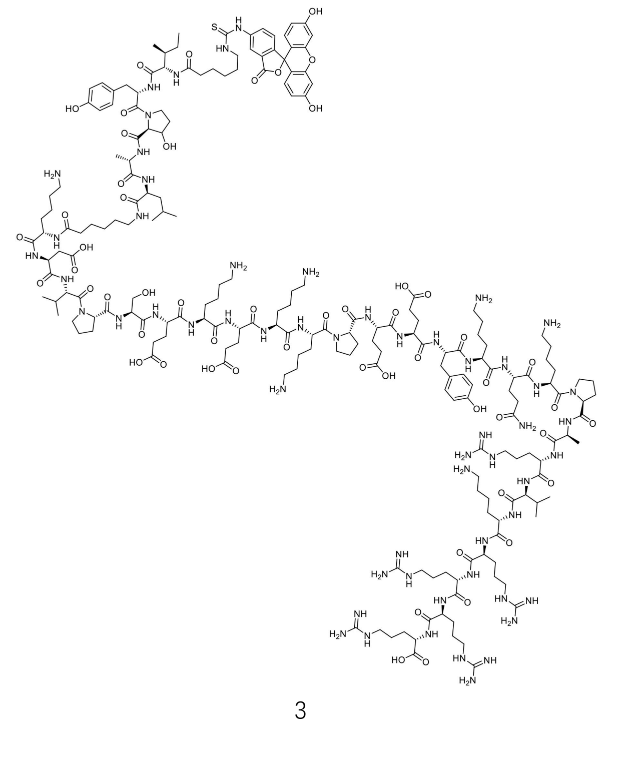

HPLC report:

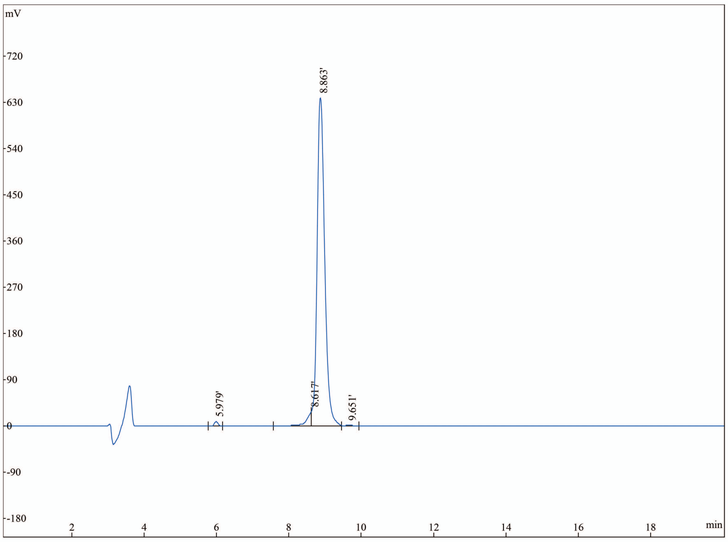

Peak No.	Ret Time	Area	Conc.
1	5.979	66676	0.7223
2	8.617	286971	3.109
3	8.863	8838102	95.74
4	9.651	39716	0.4302
**Total:**		**9231465**	**100.0000**

MS report:

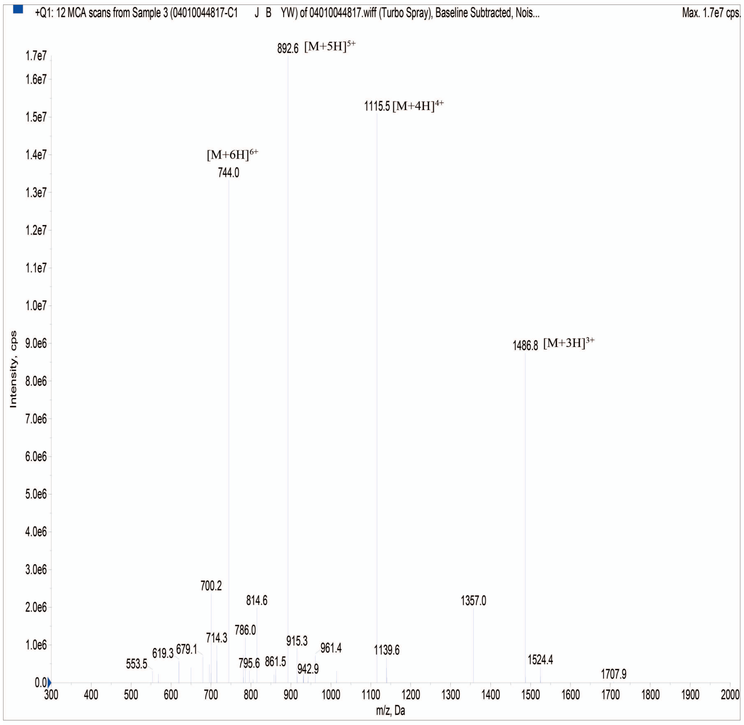

3) FITC-PRTC-v: FITC-(Ahx)-KDVLSEKEKKLEEYKQKLARVKRRRR

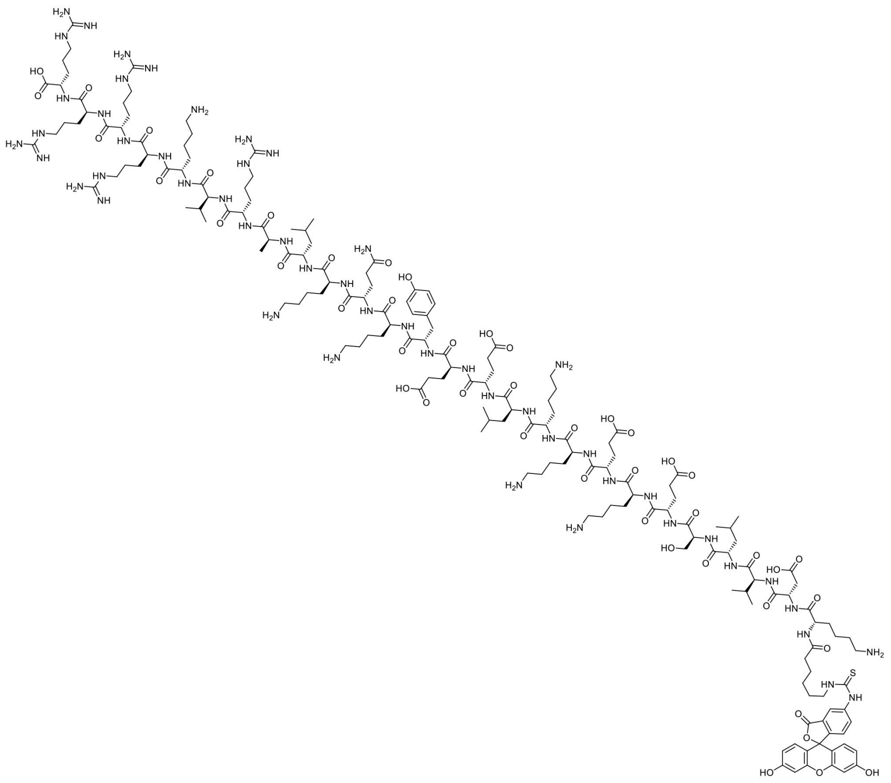

HPLC report:

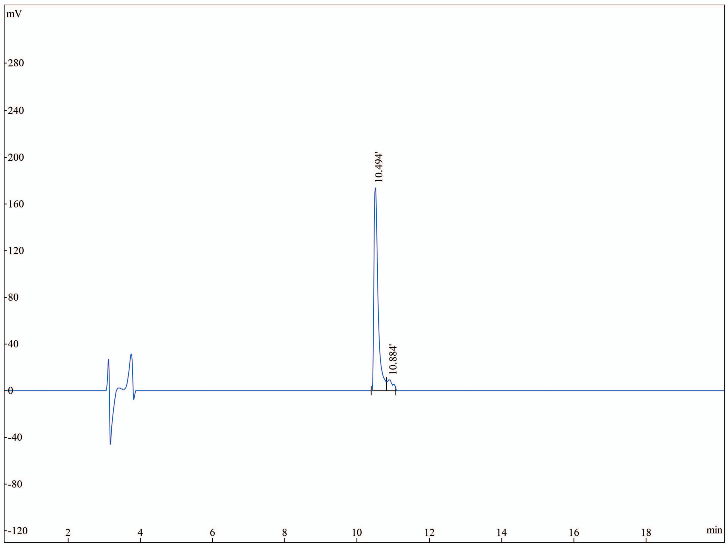

Peak No.	Ret Time	Area	Conc.
1	10.494	1263172	92.73
2	10.884	99063	7.272
**Total:**		**1362235**	**100.0000**

MS report:

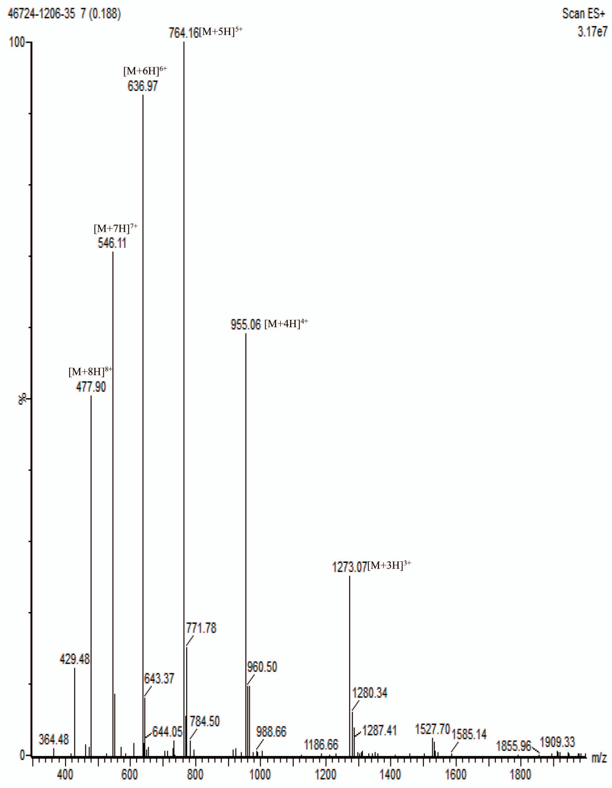

4) FITC-PRTC-r : FITC-(Ahx)-IY-(Hyp)-AL-(AHX)-KDVLSEKEKKLEEYKQKLARV

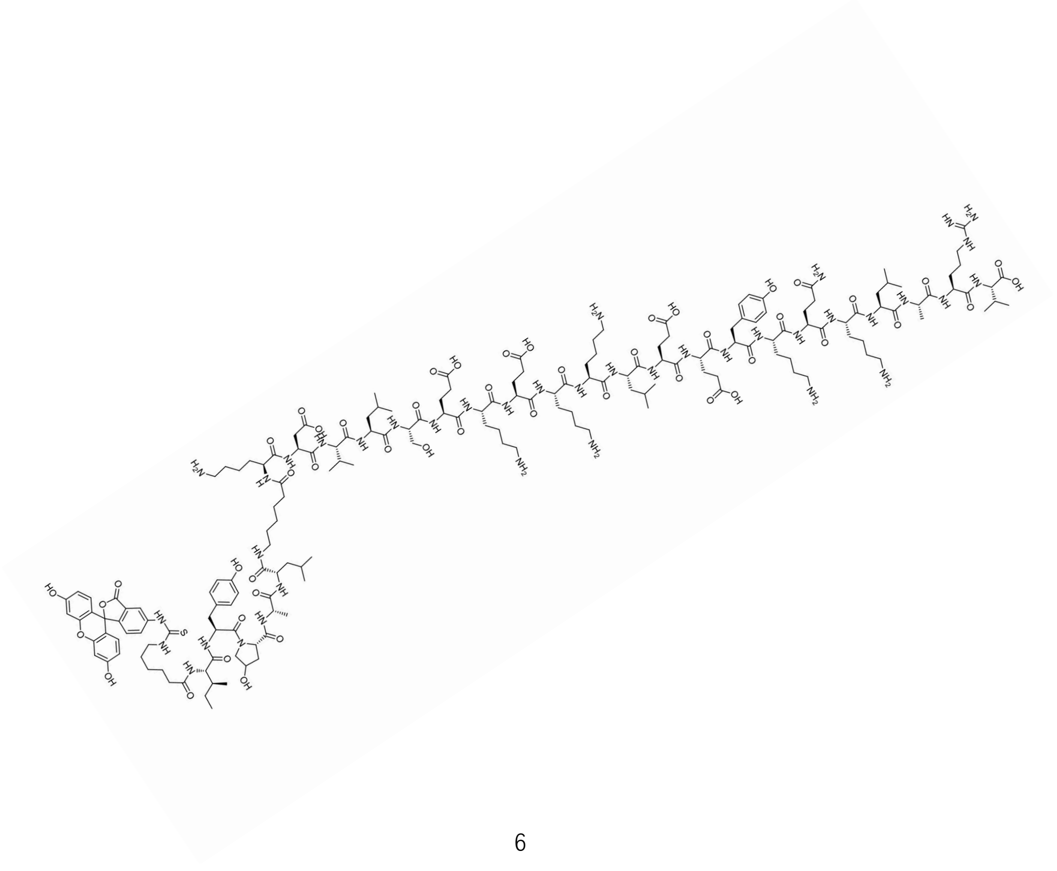

HPLC report:

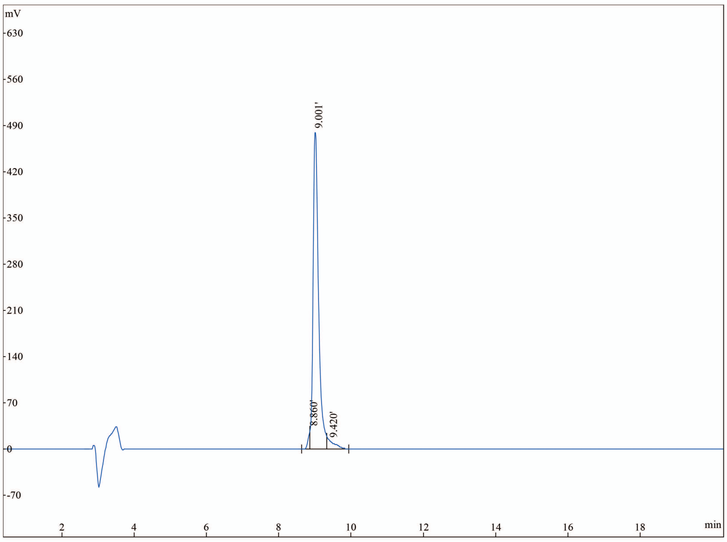

Peak No.	Ret Time	Area	Conc.
1	8.860	101984	2.052
2	9.001	4647112	93.49
3	9.420	221760	4.461
**Total:**		**4970856**	**100**

MS report:

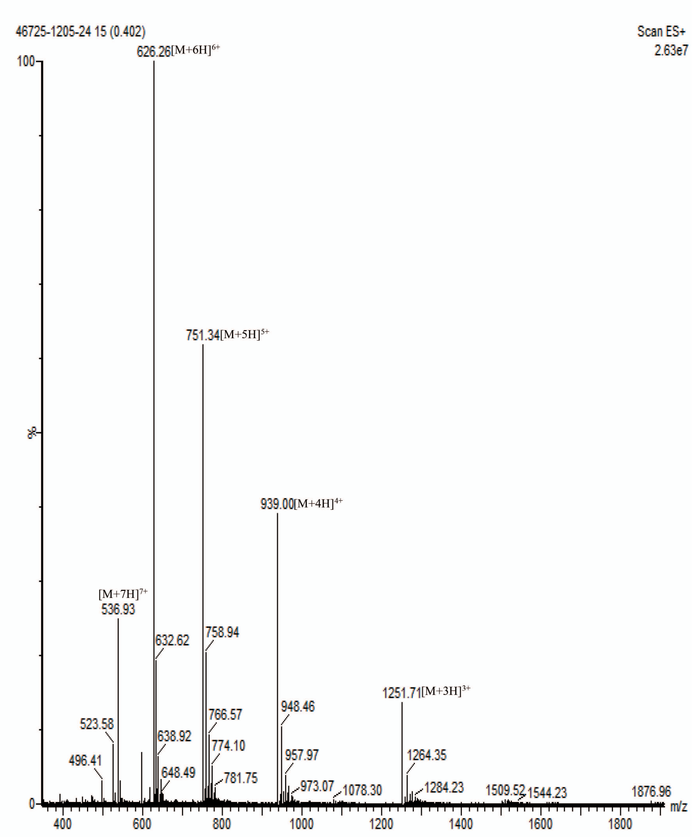

3. Supplemental table

Table S1. Analytical data of synthesized peptides

Entry	Chemical formula	Purity	MS (calculated)	MS (found)
FITC-PRTC	$C_{206}H_{333}N_{57}O_{54}S$	91.60%	4504.26	4505.76
FITC-PRTC-m	$C_{203}H_{321}N_{57}O_{54}S$	95.74%	4456.14	4458.00
FITC-PRTC-v	$C_{171}H_{279}N_{51}O_{46}S$	92.73%	3817.51	3815.80
FITC-PRTC-r	$C_{176}H_{273}N_{39}O_{49}S$	93.49%	3751.46	3751.56

4. Supplemental figures and figure legends

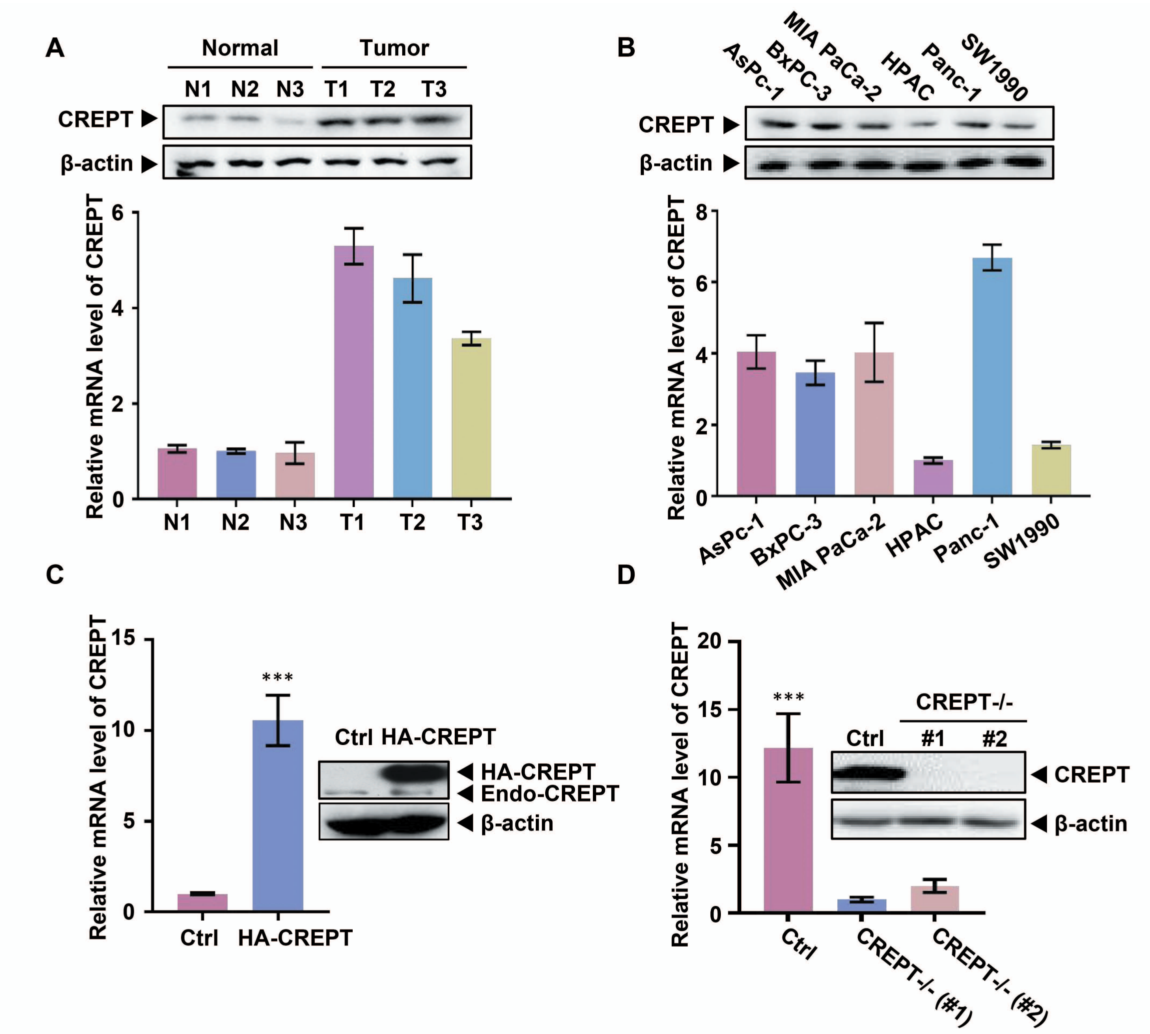

Figure S1. Overexpression of CREPT in pancreatic cancer promotes cell proliferation and tumorigenesis. (A) CREPT protein and mRNA level of mouse normal pancreatic tissues and K-ras/p53-driven pancreatic cancer tissues. N1, N2, N3 are wild type pancreatic tissues. T1, T2, T3 are tumors from KPC mouse model. **(B)** Western blot and quantitative PCR analysis of six pancreatic cell lines. **(C-D)** Identification of CREPT overexpression and CREPT deletion cell lines.

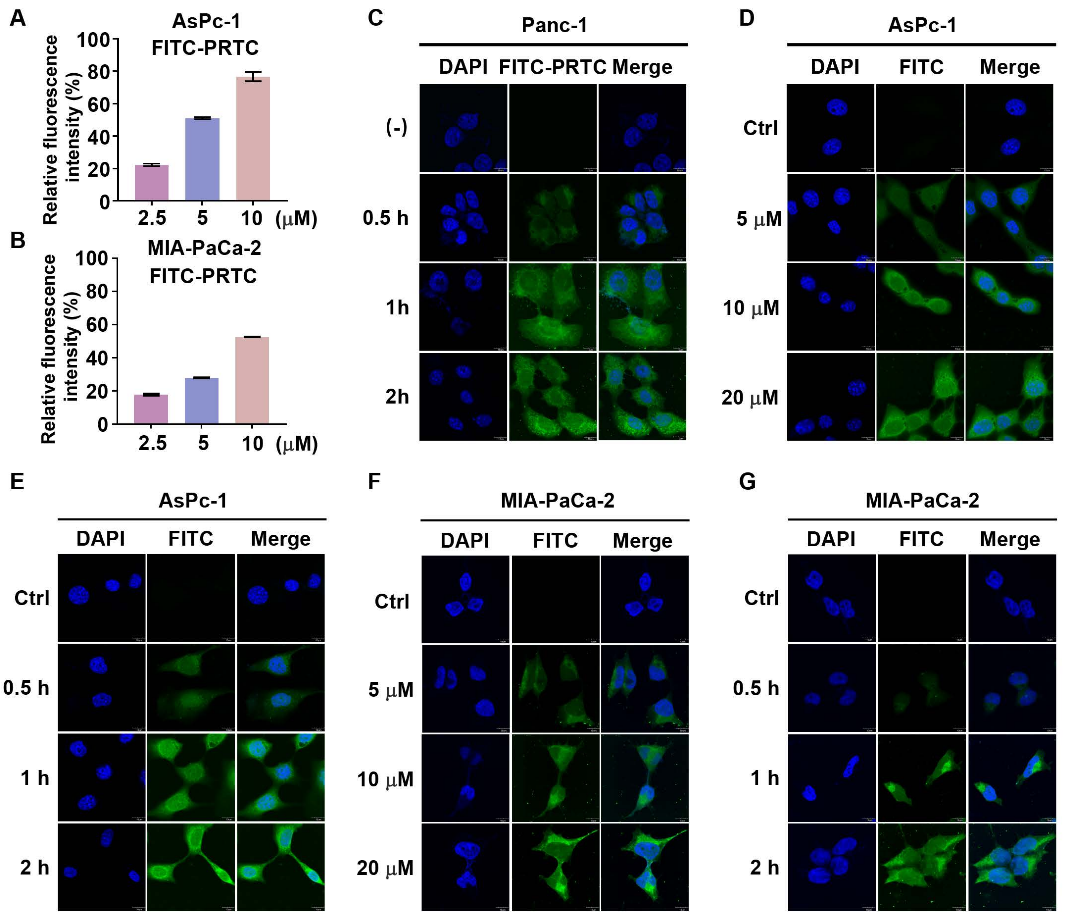

Figure S2. Identification of the permeability of PRTC in pancreatic cancer cells. (A) Quantification of cellular uptake of AsPc-1 cells treated with different concentrations of FITC-PRTC. **(B)** Quantification of cellular uptake of MIA PaCa-2 cells treated with different concentrations of FITC-PRTC. **(C)** Confocal microscope images of Panc-1 cells treated with 10 μM FITC-PRTC at different time points. Scale bars, 10 μm. **(D)** Confocal microscope images of AsPc-1 cells treated with different concentrations of FITC-PRTC. Scale bars, 10 μm. **(E)** Confocal microscope images of AsPc-1 cells treated with 10 μM FITC-PRTC at different time points. Scale bars, 10 μm. **(F)** Confocal microscope images of MIA PaCa-2 cells treated with different concentrations of FITC-PRTC. Scale bars, 10 μm. **(G)** Confocal microscope images of MIA PaCa-2 cells treated with 10 μM FITC-PRTC at different time points. Scale bars, 10 μm.

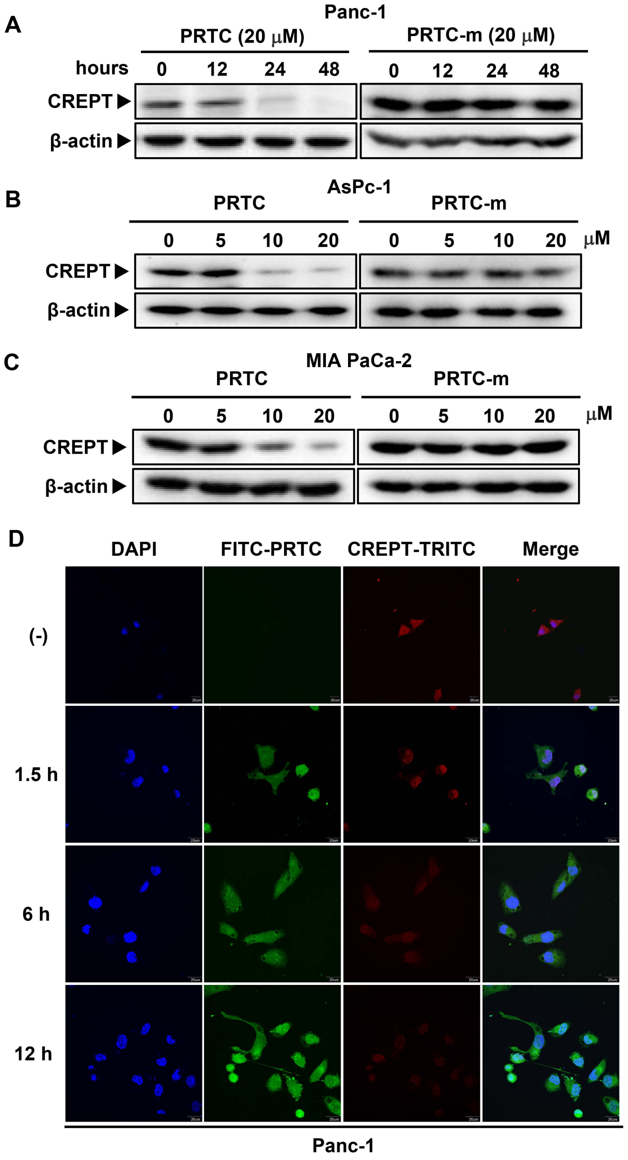

Figure S3. PRTC induces CREPT degradation. (A) Immunoblot of CREPT exposed to 20 μM PRTC or PRTC-m for different times in Panc-1 cells. **(B)** Immunoblot of CREPT following 24 h incubation with different concentrations of PRTC or PRTC-m in AsPc-1 cells. **(C)** Immunoblot of CREPT following 24 h incubation with different concentrations of PRTC or PRTC-m in MIA PaCa-2 cells. **(D)** Immunofluorescence visualization of Panc-1 cells after treatment with PRTC for 1.5 h, 6 h and 12 h. Scale bars, 20 μm.

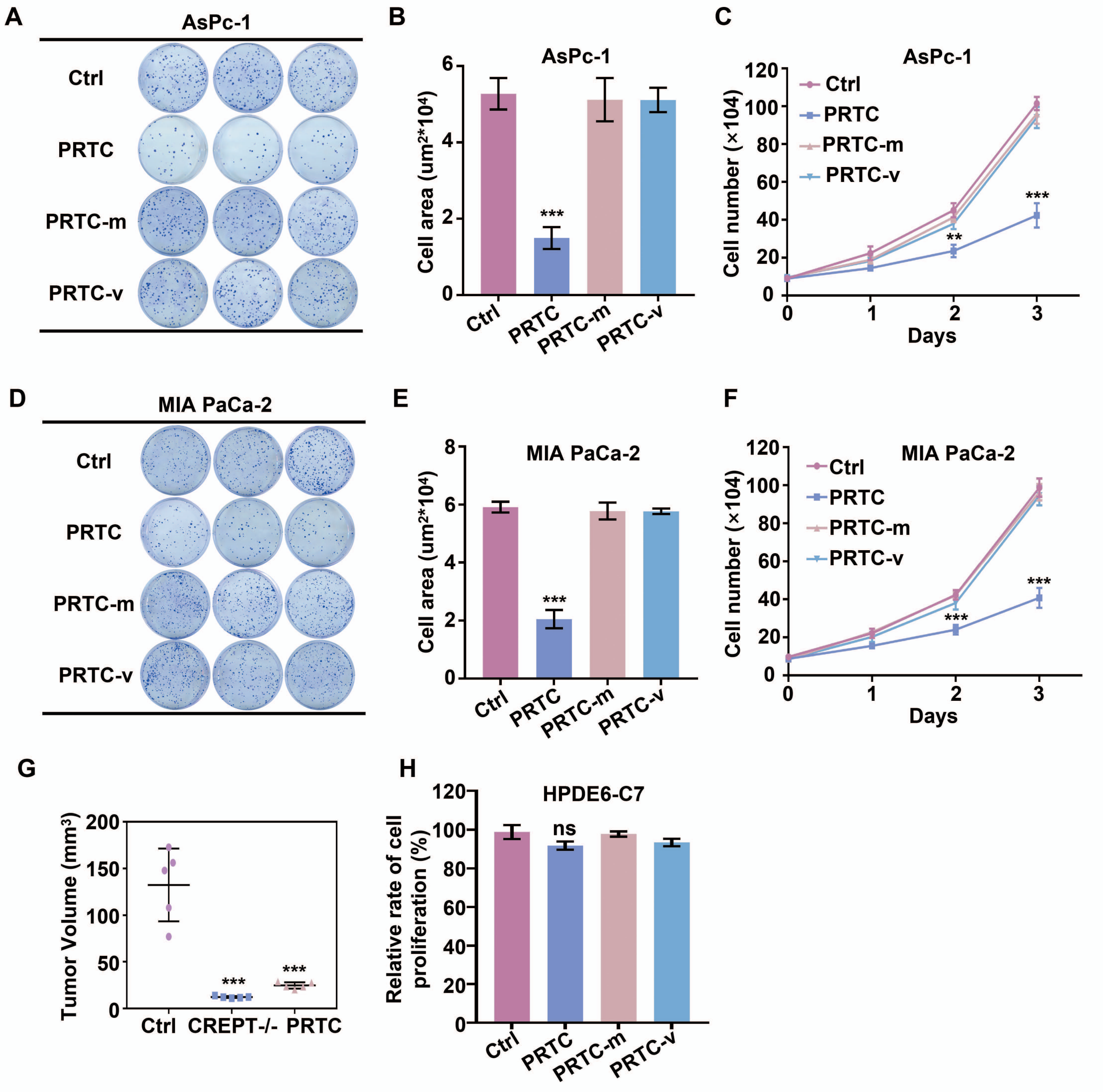

Figure S4. CREPT degradation by PRTC treatment inhibits cell proliferation and tumorigenesis in AsPc-1 and MIA PaCa-2 cells. (A-B) Colony formation of AsPc-1 respectively treated with deionized H_2O (Ctrl), 10 μM PRTC, 10 μM PRTC-m and 10 μM PRTC-v. Cells were seeded into 6-well plates (500 cells per well) and cultured for 8 days.**(C)** Cell proliferation assay of AsPc-1 cells treated with deionized water (Ctrl), 10 μM PRTC, 10 μM PRTC-m and 10 μM PRTC-v. **(D-E)** Colony formation of MIA PaCa-2 respectively treated with deionized H_2O (Ctrl), 10 μM PRTC, PRTC-m and PRTC-v. **(F)** Cell proliferation assay of MIA PaCa-2 cells treated with deionized H_2O (Ctrl), 10 μM PRTC, PRTC-m and PRTC-v. **(G)** Tumor volumes of xenograft tumors, which were measured by calipers. $V = (a \times b^2)/2$ (V is volume, a is the length of the tumor, b is the width of the tumor). **(H)** Cell proliferation assay of HPDE6-C7 cells. PRTC failed to influence the cell proliferation of HPDE6-C7 cells. HPDE6-C7 is a normal pancreatic epithelial cell line.
